# Supplementary material for: Reading instruction causes changes in category-selective visual cortex
Source: Brain Res Bull. Author manuscript; Available in PMC 2024 Jun 24. (PMC11194742; doi:10.1016/j.brainresbull.2024.110958)
Supplement: 1 [file NIHMS2000732-supplement-1.docx]

**Supplementary Information**

**Reading instruction causes changes in category-selective visual cortex**

**Sensor-space analysis of replication cohort**

To test the hypothesis that the Letter Intervention leads to an increased response to words at the expense of faces and/or objects we combined data from RCT described above with an independent replication cohort (n=16) that participated in the Letter Intervention (without random assignment). This replication cohort was originally conceptualized as a follow-up study, with a larger sample, specifically focusing on the neurobiology of reading acquisition. However, data collection was halted at the onset of the COVID-19 in March, 2020. Thus, the combined cohort included 40 participants who participated in the Letter Intervention and underwent the same MEG protocol. Individual MRI data was not available for all the participants in the replication cohort so the sensor data (n=40 participants combining original and replication cohorts) was aligned to a standard head position and statistics were calculated in sensor space.

We first examined changes in the evoked response for words by performing spatial-temporal clustering of the sensor-space data comparing the response to words post- versus pre-intervention. We found a significant increase in the response to words on left-lateralized posterior sensors spanning 300ms to 400ms after stimulus onset (**Supplementary Figure 1 top panel;** p = 0.01). Source localization (based on coregistering each participant’s data directory to the fsaverage template) indicated that the effect was localized to VOTC (in the vicinity of the VWFA), lateral temporal cortex (superior temporal sulcus and middle temporal gyrus) and inferior parietal cortex (**Supplementary Figure 1 middle panel**). The same analysis for faces and cars did not reveal any significant changes between the pre- and post-intervention data. Thus, any pruning of the response to faces and/or cars was not large enough and consistent enough to survive a spatial-temporal clustering correction for multiple comparisons. Since the time-window identified based on spatial-temporal clustering of the sensor-space data was later than the time-window with the largest intervention effect in the source-space data we next ran an analysis restricted to posterior, left-lateralized sensors and the time window of 135-235ms and found a significant increase in the response to words (p < 0.05).


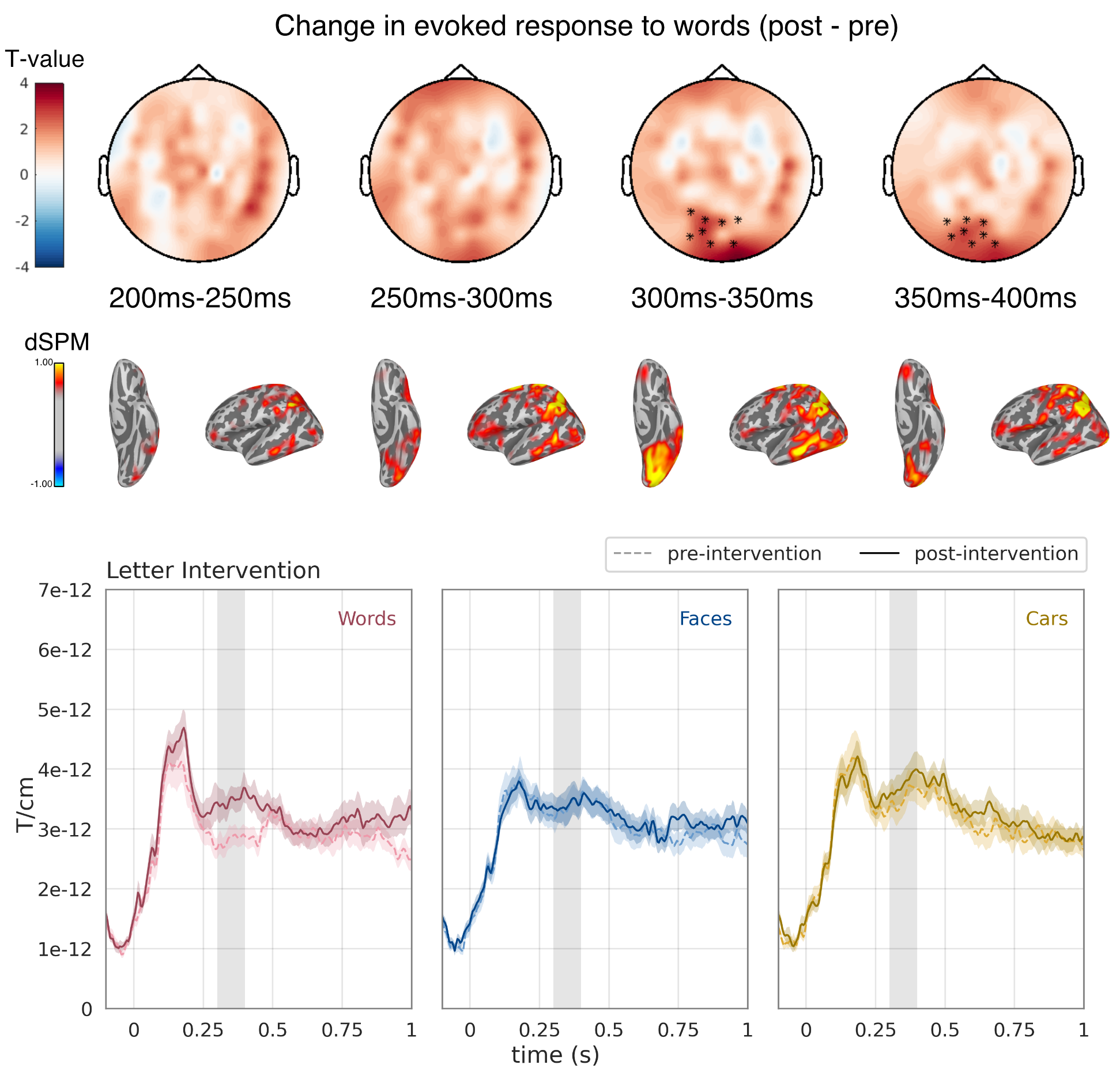


***Supplementary Figure 1: Enhanced response to words after the Letter Intervention.*** *Topo plots show t-statistics comparing the response to words post- versus pre-intervention. Warm colors indicate an increase in response and asterisks indicate the cluster of sensors and timepoints that were significant based on spatiotemporal clustering of the sensor space data. Source estimates (dSPM values) are shown below with warm colors indicating an increased response to words after the letter intervention. The evoked response to Words, Faces and Cars is shown for the cluster of sensors with a significant intervention effect (note that plots are shown for sensors that were identified as significant based on spatiotemporal clustering meaning that effects illustrated in the figure are not independent of sensor selection). Gray shading indicates the significant time window identified based on spatiotemporal clustering.*

To more directly examine competition, we created a ROI encompassing the cluster of sensors showing a significant increase in the evoked response to words (shown in **Supplementary Figure 1**). We then extracted the average evoked response within this ROI for each condition and time point and calculated the correlation between the change in response to words versus other categories. A negative correlation would indicate that the increase in word response was coupled to a decrease in the response to another category. We first examined the average response within the time window corresponding to the significant increase in the word response (300-400ms) and found a significant positive correlation for words and faces (r=+0.46, p=0.005) and a positive correlation that was not significant for words and cars (r=+0.27, p=0.11). We next conducted an exploratory analysis of 50ms time windows spanning 100ms to 400ms to ensure we hadn’t missed a time-localized negative correlation. For each time window there was a significant positive correlation between the intervention-driven change in the response to words and faces (+0.52< r <+0.65 , 0.000015 < p <0.001). The correlation between the change in response to words and cars was positive at each time-point but was only marginally significant (+0.12< r <+0.37 , 0.03< p <0.48 ). Thus, the increase in the response to words was not coupled to a decrease in the response to any other category within this ROI.

Supplementary Table 1: Detailed Schedule for a day of Letter Intervention

| Time | Activity | Example |
| --- | --- | --- |
| 15 minutes | Free Play | puzzles, blocks, playdough |
| 25 minutes | Phonemic Awareness | Song, games, chant, etc. |
| 25 minutes | Recess | Free play outside |
| 4 minutes | Direct instruction part 1 (new letter) | Teacher introduces letter picture card.  Teacher models letter formation on white board.  Students individually trace letter at white board. |
| 3 minutes | letter formation practice | Students trace letter on 11X17 paper using 2 fingers, eraser, and pencil tip. |
| 3 minutes | phoneme grapheme correspondence | Teacher introduces key word and letter sound.  Teacher and students air write letter and say its “name, key word and sound”  Class reviews letter flashcards |
| 5 minutes | decoding | Students decode a cvc word at the white board with teacher assistance |
| 25 min | center rotation | games, coloring, tracing, sorting, matching, etc. |
| 25 minute | snack and recess | free play outside |
| 4 minutes | Direct instruction part 2 (new letter) | Teacher introduces letter picture card.  Teacher models letter formation on white board.  Students individually trace letter at white board. |
| 3 minutes | letter formation practice | Students trace letter on 11X 17 paper using 2 fingers, eraser, and pencil tip. |
| 3 minutes | phoneme grapheme correspondence | Teacher introduces key word and letter sound.  Teacher and students air write letter and say its “name, key word and sound”  Class reviews letter flashcards |
| 5 minutes | decoding | Students decode a cvc word at the white board with teacher assistance |
| 25 min | center rotation | games, coloring, tracing, sorting, matching, etc. |
| 10 minutes | read aloud | Teacher reads story to class |

Supplementary Table 2: Detailed Schedule for a day of Language Intervention

| Time | Activity | Example |
| --- | --- | --- |
| 15 minutes | Free Play | puzzles, blocks, playdough |
| 25 minutes | Syntax Instruction | Noun, verb, plural, tense |
| 25 minutes | Recess | free play outside |
| 5 minutes | Read aloud | Narrative Text |
| 10 minutes | Narrative Text Instruction | Character, setting, etc. |
| 25 minutes | Center Rotation | games, coloring, tracing, sorting, matching, etc. |
| 25 minutes | Snack and Recess | free play outside |
| 15 minutes | Semantic Feature Analysis Instruction | Colors, shapes, jobs, clothing, etc. |
| 25 min | Center rotation | Games, coloring, tracing, sorting, matching, etc. |
| 10 minutes | read aloud | Teacher reads story to class |
